# Disseminated Tuberculosis Associated Hemophagocytic Lymphohistiocytosis in a Pregnant Woman With Evans syndrome: A Case Report and Literature Review

**DOI:** 10.3389/fimmu.2021.676132

**Published:** 2021-06-10

**Authors:** Yun-Feng Shi, Xiao-Han Shi, Yuan Zhang, Jun-Xian Chen, Wen-Xing Lai, Jin-Mei Luo, Jun-Hui Ba, Yan-Hong Wang, Jian-Ning Chen, Ben-Quan Wu

**Affiliations:** ^1^ Medical Intensive Care Unit, Department of Respiratory and Critical Care Medicine, Third Affiliated Hospital of Sun Yat-Sen University, Guangzhou, China; ^2^ Institute of Respiratory Diseases, Sun Yat-Sen University, Guangzhou, China; ^3^ Department of Gynaecology and Obstetrics, Third Affiliated Hospital of Sun Yat-Sen University, Guangzhou, China; ^4^ Department of Hematology, Third Affiliated Hospital of Sun Yat-Sen University, Guangzhou, China; ^5^ Department of Pathology, Third Affiliated Hospital of Sun Yat-Sen University, Guangzhou, China

**Keywords:** tuberculosis, hemophagocytic lymphohistiocytosis, Evans syndrome, corticosteroid, case report

## Abstract

**Background:**

Tuberculosis (TB) is a leading cause of morbidity and mortality in underdeveloped and developing countries. Disseminated TB may induce uncommon and potentially fatal secondary hemophagocytic lymphohistiocytosis (HLH). Timely treatment with anti-tuberculosis therapy (ATT) and downmodulation of the immune response is critical. However, corticosteroid treatment for TB-associated HLH remains controversial. Herein, we report a successful case of disseminated TB-associated HLH in a pregnant woman with Evans syndrome accompanied by a literature review.

**Case Presentation:**

A 26-year-old pregnant woman with Evans syndrome was transferred to the Third Affiliated Hospital of Sun Yat-Sen University because of severe pneumonia. She presented with cough, fever, and aggravated dyspnea. Nested polymerase chain reaction for *Mycobacterium tuberculosis* (*M. tuberculosis*) complex in sputum was positive. Sputum smear sample for acid-fast bacilli was also positive. Metagenome next-generation sequencing (mNGS) of the bronchoalveolar lavage fluid identified 926 DNA sequence reads and 195 RNA sequence reads corresponding to *M. tuberculosis* complex, respectively. mNGS of blood identified 48 DNA sequence reads corresponding to *M. tuberculosis*. There was no sequence read corresponding to other potential pathogens. She was initially administered standard ATT together with a low dose of methylprednisolone (40 mg/day). However, her condition deteriorated rapidly with high fever, acute respiratory distress syndrome, pancytopenia, and hyperferritinemia. Bone marrow smears showed hemophagocytosis. And caseating tuberculous granulomas were found in the placenta. A diagnosis of disseminated TB-associated HLH was made. Along with the continuation of four drug ATT regimen, therapy with a higher dose of methylprednisolone (160 mg/day) combined with immunoglobulin and plasma exchange was managed. The patient’s condition improved, and she was discharged on day 19. Her condition was good at follow-up with the continuation of the ATT.

**Conclusions:**

Clinicians encountering patients with suspected TB accompanied by unexplainable inflammation not responding to ATT should consider complications with HLH. Timely administration of ATT combined with corticosteroids may result in a favorable outcome.

## Introduction

Hemophagocytic lymphohistiocytosis (HLH), also known as a hemophagocytic syndrome, is a life-threatening hyperinflammatory syndrome caused by uncontrolled activation of cytotoxic T lymphocytes, natural killer (NK) cells, and macrophages, resulting in hypercytokinemia and immune-mediated injury in multiple organs (1, 2). As a syndrome, the clinical manifestations of HLH vary, including fever, organomegaly, cytopenia, consumptive coagulopathy, hypertriglyceridemia, and elevation of acute-phase reactants (2). HLH is generally classified as primary or secondary. Secondary HLH is usually induced by an infection, autoimmune disease, or malignancy (2, 3). *Mycobacterium tuberculosis* (*M. tuberculosis*) infection-induced HLH is uncommon and fatal, with approximately 3% morbidity and 49% mortality (4). For disseminated tuberculosis (TB)-associated HLH, diagnosis is difficult, and the treatment is challenging (5, 6). Although the number of reported TB-associated HLH has been increasing in recent years, survival in complicated case is rare (3, 7). Here, we present a case of disseminated TB-associated HLH in a pregnant woman with Evans syndrome, who achieved a favorable outcome following targeted therapy with antitubercular medications and corticosteroids. Literature retrieval found no such case published before. It is worth reporting and publishing such complicated and rare case of disseminated TB-associated HLH in a pregnant woman with Evans syndrome.

## Case Presentation

A 26-year-old pregnant woman was admitted owing to cough for 11 days, fever for 6 days and dyspnea for 2 days at 15 weeks of gestation. She presented with a dry cough 11 days prior to admission, accompanied by profound fatigue and night sweats. After treatment with Chinese patent medicine, the symptoms were temporarily resolved. High fever occurred 6 days prior, with a peak temperature of 39.3°C, accompanied by cough with white sputum. There was no clinical response to symptomatic treatment administered. Her previous symptoms aggravated 2 days prior, with a peak temperature of 40.1°C, along with dyspnea and altered sensorium. There were no chills, loss of consciousness, hemoptysis, or colporrhagia. Chest X-ray showed diffuse bilateral infiltration and nested polymerase chain reaction (PCR) for *M. tuberculosis* complex in sputum was positive, which was performed in the Maternal and Child Health Hospital. She was then transferred to the medical intensive care unit (MICU) of the Third Affiliated Hospital of Sun Yat-Sen University for isolation and further treatment.

The patient had a past history of Evans syndrome (ES) for 4 years. She regularly took oral corticosteroids (prednisone 20-45mg/day) and had normal hemoglobin and platelet counts. She had no disorders related to the other organs and was a non-smoker. She had no past history of *M. tuberculosis* infection and any recent travel, tick bites, or poultry contact. Her pregnancy duration was uneventful to date.

On admission to the MICU (day 1), her vital signs were as follows: body temperature, 37.0°C, pulse rate, 120 beats/min, respiratory rate, 40 breaths/min, blood pressure, 140/68 mmHg, and oxygen saturation, 95.0% with breathing 15 L/min of oxygen by venture mask. She was drowsy and appeared to be acutely ill. The diminished breath sounds and wet rales in both lungs were heard on auscultation. There was no audible murmur on cardiac auscultation. Tenderness, hepatomegaly and splenomegaly were not detected. The patient had no neck stiffness. No rash was observed.

**Figure 1:**
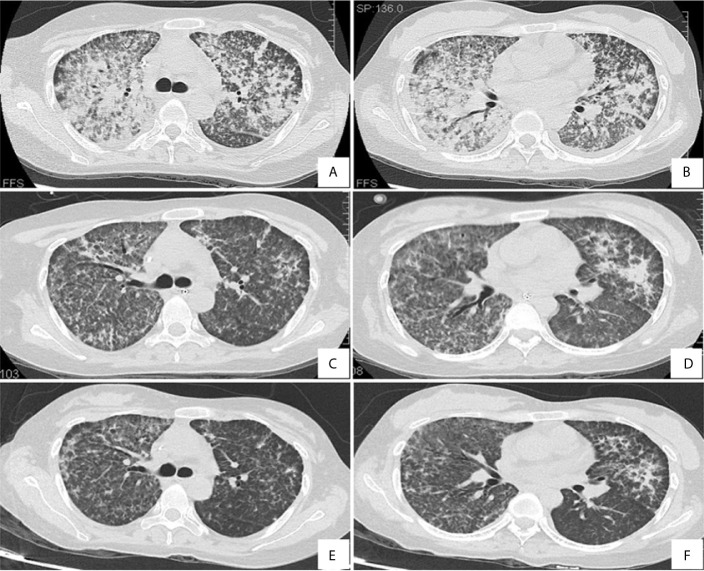
Chest computed tomography scan on day 2 **(A, B)**, day 8 **(C, D)** and day 17 **(E, F)**.

Laboratory data upon admission revealed a white blood cell (WBC) count of 13.86 × 10^9^/L with an elevated neutrophil ratio of 94.0% and normal eosinophil ratio, hemoglobin of 101 g/L, and platelet count of 73 × 10^9^/L. The concentration of C-reactive protein (CRP) and procalcitonin was 222.0mg/L and 3.29ng/mL, respectively. The erythrocyte sedimentation rate (ESR) was 47 mm/h. Albumin was 20.8g/L. Globulin was 21.8g/L. Triglyceride level was 1.3 mmol/L. Her lactic dehydrogenase (LDH) level was 568 U/L. The uric acid concentration was 429 µmol/L. Electrolytes, creatinine, aspartate aminotransferase, alanine aminotransferase, total bilirubin and direct bilirubin levels were within normal limits. Even with continuous nasal high flow oxygen therapy, arterial blood gas analysis showed a pH of 7.44, PO_2_ of 71.0 mmHg, PCO_2_ of 38 mmHg, oxygenation index of 87.6, and lactate level of 1.0 mmol/L. Coagulation tests demonstrated prothrombin time of 13.7 s, prothrombin activity of 95%, and fibrinogen 5.0 g/L. Serology for respiratory viruses (adenovirus, influenza A virus, influenza B virus, parainfluenza, and respiratory syncytial virus) as well as bacteriological assays (Mycoplasma pneumoniae, Chlamydia pneumonia, and *Legionella*) were all negative. Tests for cytomegalovirus, Epstein Barr virus, *Toxoplasma*, rubella virus, herpes simplex virus, HIV, hepatitis A, B, C, and E viruses, dengue virus, malaria, *Leptospira*, and scrub typhus were also negative. Cryptococcal antigen was negative. The galactomannan and β-D-glucan tests were both negative. The interferon-gamma release assay was indeterminate. Peripheral blood culture was negative. However, the sputum smear sample was positive for acid-fast bacilli. All the indicators were negative for routine laboratory screening (anti-nuclear antibody, anti-extractable nuclear antigen antibody, and anti-neutrophilic cytoplasmic antibody, et al) for autoimmune diseases. Coombs test was negative (The detailed immunological profile was shown in **Supplementary File 1**). The counts of CD3, CD4, and CD8 T lymphocytes in blood were 520/mm^3^, 208/mm^3^ and 284/mm^3^ respectively, with a decreased CD4/CD8 ratio of 0.73. After admission, a lumbar puncture showed no abnormalities. There was no evidence in cytology and biochemistry of cerebrospinal fluid supporting the existence of tuberculous meningitis. Chest computed tomography (CT) revealed diffuse infiltration and patchy shadows in both lungs and bilateral pleural effusion (**Figures 1A, B**). Abdominal CT revealed splenomegaly but showed no peritonitis and seroperitoneum.

**Figure 2:**
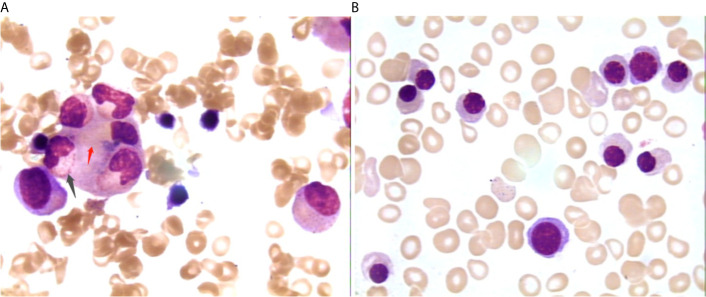
Changes of bone marrow smear before **(A)** and after **(B)** treatment 1000×. Picture A showing neutrophils (black arrow) and red cells (red arrow) engulfed by macrophages; picture B showing no phagocytosis.

**Figure 3:**
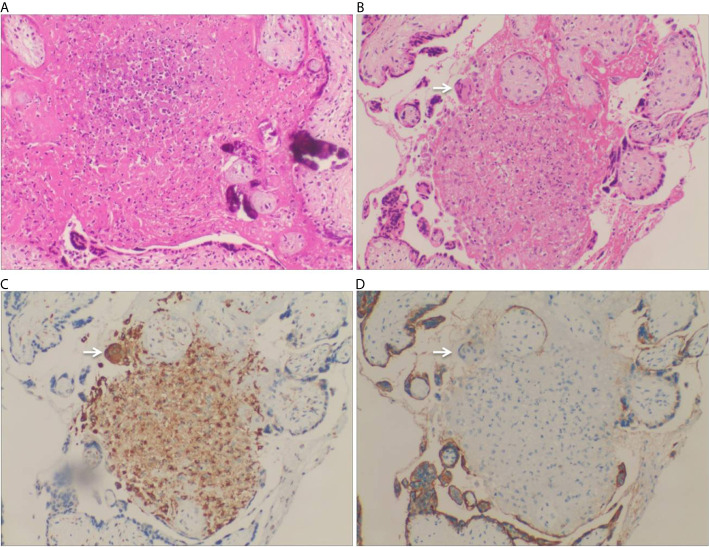
Pathology of placenta biopsy showing caseating tuberculous granulomas. **(A)** Caseous necrosis in a placenta (H&E staining, 200×). **(B)** Granuloma was found in the placenta, which was comprised of proliferative epithelioid cells. A Langhans multinucleated giant cells was found at the edge of the nodule (arrow) (H&E staining, 200×). **(C)** CD68 immunohistochemical staining highlighted the proliferative epithelioid cells as well as the Langhans multinucleated giant cell (CD68 immunostaining, 200×). **(D)** The proliferative epithelioid cells and the Langhans multinucleated giant cell were negative for PLAP, while the placental syncytiotrophoblast cells were positive for PLAP (PLAP immunostaining, 200×).

After admission, the patient was initially administered with non-invasive positive pressure ventilation, anti-tuberculosis therapy (ATT), and a low dose of methylprednisolone (40 mg qd intravenous infusion). The details of ATT were isoniazid (0.8 g qd intravenous infusion), rifampin (0.4 g qd orally), ethambutol (0.75 g qd orally), pyrazinamide (0.5 g TID orally) and moxifloxacin (0.4 g qd intravenous infusion). However, the patient’s condition deteriorated rapidly with high fever, acute respiratory distress syndrome (ARDS), pancytopenia, and hyperferritinemia on day 3. The patient was treated with tracheal intubation and mechanical ventilation. Metagenome next-generation sequencing (mNGS) of the bronchoalveolar lavage fluid (BALF) identified 926 of 225958 DNA sequence reads and 195 of 491668 RNA sequence reads corresponding to *M. tuberculosis* complex, respectively. mNGS of blood identified 48 of 125859 DNA sequence reads corresponding to *M. tuberculosis*. There was no sequence read corresponding to other potential pathogens (Methods, quality control and detailed results of mNGS were shown in **Supplementary File 2**). Bone marrow smears showed increased macrophage activity with hemophagocytosis without evidence of leukemia or lymphoma. And there was no evidence of *M. tuberculosis* in bone marrow (**Figure 2A**). Additional blood tests showed hyperferritinemia (6649 ng/mL, normal range 10-291 ng/mL), low NK cell activity (13.08%, normal value: ≥15.1%, tested by flow cytometry) and elevated soluble CD25 levels (5730 pg/mL, normal value: <2400 pg/mL, tested by ELISA). Genetic testing for primary HLH was negative (Detailed result of genetic testing was shown in **Supplementary File 3**). As the embryo died on day 5, she underwent a medical abortion, and caseating tuberculous granulomas were found in the placenta (**Figure 3**). Clinical laboratory tests and imaging were performed by the department of laboratory and image center of the Third Affiliated Hospital of Sun Yat-sen University, respectively. mNGS, NK cell activity, level of soluble CD25 and genetic testing for primary HLH were performed and reported by Guangzhou Kingmed Medical Test Center Co., Ltd.

**Figure 4:**
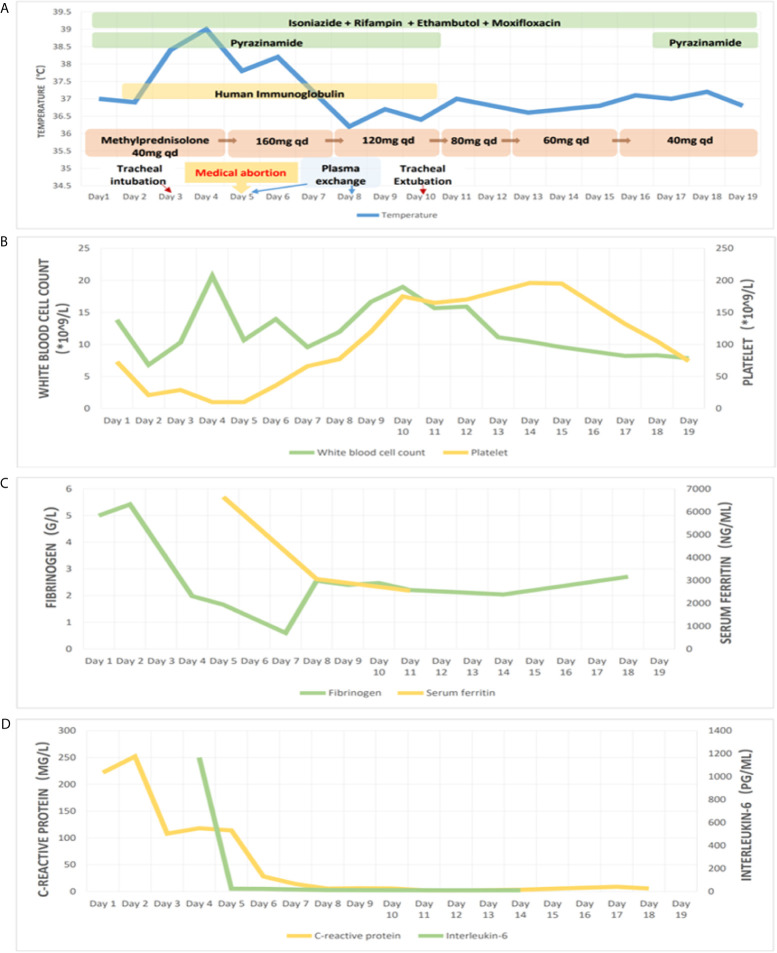
Treatment and observation during hospitalization. Picture **(A)** showing body temperature and treatment; picture **(B)** showing white blood cell count and platelet count; picture **(C)** showing serum fibrinogen and ferritin; picture **(D)** showing C-reactive protein and interleukin-6, during hospitalization.

At the most severe moment, the patient was assessed with an acute physiology and chronic health evaluation (APACHE) II score of 32, sepsis related organ failure assessment (SOFA) score of 14, Marshall score of 14 and mortality risk of 85.3%. Following consultation with a multidisciplinary team, a diagnosis of disseminated TB-associated HLH was made. Along with the continuation of four drug ATT regimen, therapy of higher dose of methylprednisolone (80 mg bid intravenous infusion) combined with immunoglobulin (20 g qd intravenous infusion, days 2-10) and plasma exchange (2000 mL each time on days 4 and 8) was administered. The patient responded well to the treatment, with marked improvement in fever and cytopenia (**Figures 4A, B**). She failed to respond to transfusion of platelet initially, then platelet count began to increase spontaneously after combined therapeutic strategy. Elevation of eosinophil ratio was not noted during the treatment. Along with the resolution of hypofibrinogenemia and hyperferritinemia (**Figure 4C**), the concentrations of CRP and interleukin (IL)-6 decreased continuously (**Figure 4D**). The second bone marrow aspiration showed no hemophagocytosis (**Figure 2B**). Her condition improved daily, along with the increase in oxygenation index and improvement in chest CT gradually (**Figures 1C–F**); she was withdrawn from the ventilator on day 10 and was discharged on day 19. Two weeks later, pleural fluid culture revealed *M. tuberculosis* and was confirmed by Xpert.

Based on the clinical data, the patient was finally diagnosed with disseminated TB-associated HLH, severe pneumonia, type I respiratory failure, severe ARDS, medical abortion, and ES.

The patient was in good condition during the follow-up. Two months after discharge, the standard four-drug ATT was changed to a two-drug continuation phase with isoniazid and rifampin. Methylprednisolone was reduced to 12 mg qd orally. No significant abnormalities were found in WBC, hemoglobin, platelet count, CRP, ESR, LDH, ferritin, and uric acid in the serum.

## Discussion

Disseminated TB can cause systemic inflammatory response syndrome, which may lead to fatal HLH. Due to low awareness and variable clinical manifestations of the disease, disseminated TB-associated HLH is often not recognized by clinicians. Early treatment may lead to improved survival. However, the utility of immunosuppressive therapy lacks a general consensus. Therefore, it is necessary to discuss and review the disease to improve clinical awareness and management.

The lack of specific clinical manifestations, low sensitivity for acid-fast staining, and time-consuming culture make the diagnosis of disseminated tuberculosis difficult, especially in severe cases (8). Chest CT does not always show a typical miliary nodular pattern. Definitive diagnosis depends on the isolation of *M. tuberculosis* by culture or the identification of specific sequences of DNA by PCR or mNGS in related samples (9–12). In the present case, the nucleic acid of *M. tuberculosis* complex in sputum and BALF was detected by PCR and mNGS, respectively. The nucleic acid of *M. tuberculosis* in peripheral blood and the culture of *M. tuberculosis* in the pleural fluid were confirmed. In addition, although there was no evidence of tuberculous meningitis and tuberculous peritonitis, caseating tuberculous granulomas were found in the placenta. The etiologic diagnosis of disseminated TB was made according to above clinical data.

Extensive research shows that pancytopenia in disseminated TB suggests the risk of HLH (4, 8, 13). HLH is an uncommon but fatal complication of disseminated TB. The diagnosis of secondary HLH is established by fulfilling the HLH 2004 criteria, which is any five out of the following eight criteria: prolonged fever; splenomegaly; unexplained progressive cytopenia (affecting ≥2 of 3 lineages in the peripheral blood, the standard is hemoglobin <90 g/L, platelet count <100 × 10^9^/L, absolute neutrophil count <1 × 10^9^/L); hypertriglyceridemia and/or hypofibrinogenemia (fasting triglycerides ≥3.0 mmol/L, fibrinogen ≤1.5 g/L); hemophagocytosis in the bone marrow, spleen, or lymph nodes without evidence of malignancy; hyperferritinemia (≥500 ng/mL); low or absent of NK-cell activity; and elevated soluble CD25 levels (≥2400 IU/ML of soluble IL-2 receptor) (1, 14, 15). Our patient met all of the criteria outlined above, along with the elimination of primary HLH, therefore a final diagnosis of disseminated TB-associated HLH was established. High APACHE II score, SOFA score, Marshall score and mortality risk indicated the severity of our patient. As the diagnosis is confirmed and severity is assessed, treatment is critical for survival.

In addition to eliminating the originated causes of HLH, treatment directed at HLH is downmodulating the immune response (1, 6, 16, 17). Immunosuppressive therapies include the use of corticosteroids, etoposide and cyclosporine A (CSA). Corticosteroids are believed to be the basal agents in the HLH-1994 and HLH-2004 treatment protocols. In the HLH-2004 protocol, reduced use of etoposide and CSA was recommended in adult and elderly patients because of their side effects (6). Immunomodulatory therapy, including intravenous immunoglobulin (IVIG) and plasma exchange, is regarded as an effective and safe method for severe cases with multiple organ dysfunction syndrome (MODS), such as this patient (6, 18, 19).

Management of tuberculosis is critical in disseminated TB-associated HLH. Standard four-drug ATT and second-line fluoroquinolone drugs have been proven to be effective against TB (9, 20). However, in the present patient, the initial ATT seemed to be ineffective because her condition deteriorated during treatment. Such life-threatening conditions may require more intensive combination treatment with ATT, such as corticosteroids and immunomodulatory therapy. Crichley et al. reported that corticosteroid therapy reduced the mortality rate by 17% for all forms of TB (21). In 2006, Brastianos et al. reported that approximately 68% of cases of TB-associated HLH received a combination of ATT and immunomodulatory treatment (mainly corticosteroids), and 60% of them survived (22). In a literature review for the past 5 years, approximately 60% of TB associated-HLH cases were treated with corticosteroids (23–26). Corticosteroid has also been applied to more severe nontuberculosis mycobacterium infection-associated HLH (27). Some severe cases of TB-associated HLH treated without corticosteroid led to patient mortality (3, 28, 29). So, although corticosteroid treatment in TB-associated HLH remains debatable, more evidence support the role of corticosteroid in severe or complicated cases. Just as what Padhi et al. concluded, the determination of treatment priorities would be based on the clinical condition of the patient (4). In patients with complication of sepsis or MODS, corticosteroids remain the foundation of early management.

In addition, TB-associated HLH occurs more frequently in patients with underlying autoimmune diseases such as ES as in the present case, Poncet’s disease, or with other comorbidities, which accounts a total proportion as high as 65% (4, 7, 28, 30). ES is a chronic autoimmune disease characterized by the concomitant or sequential association of hemolytic anemia with thrombocytopenia. The treatment of ES mostly relies on corticosteroids (31, 32). Therefore, corticosteroid treatment is essential for this patient, even just for the underlying ES. There is few study and article concerning ES and HLH. But according to the principle of pathology, it suggests that the link of ES and HLH is not just they both are autoimmune diseases, but also lies on infection. The long-term corticosteroid treatment would lead to immunosuppressive condition. Just as in the present patient, most cases with ES present a decreased CD4/CD8 ratio, which indicates the suppression of immunity (31). Immunosuppression may result in opportunistic infection, such as tuberculosis, which is likely to induce HLH. The onset of TB associated HLH, together with the underlying autoimmune diseases, both requires corticosteroid and/or immunomodulatory therapy.

As for the suitable dose of corticosteroid in disseminated TB-associated HLH, no consensus has been reached. In our patient, when a low dose of corticosteroid seemed to be insufficient, a higher dose of corticosteroid was required and received an encouraging clinical response. A study reported a case of tuberculosis associated HLH cured by steroid pulse therapy (intravenous methylprednisolone 1000 mg/d for three days) (33). However, physicians are more concerned about the side effect of corticosteroids. Corticosteroids induce a wide immunosuppressive effect by inhibiting the activation of immunocytes and suppressing the production of inflammatory cytokines, which might aggravate tuberculosis infection. Nevertheless, this risk could be maximally eliminated by sufficient anti-infection therapy together with close observation, careful monitoring, and accurate assessment of patients’ condition or organ function. For example, in addition to body temperature, inflammatory cytokines and chest CT are considered objective markers for inflammation and the effectiveness of treatment. As presented in our patient, with an early confirmed diagnosis, sufficient ATT, and careful monitoring of the inflammation, the use of appropriate dose of corticosteroid would be beneficial and safe in disseminated TB-associated HLH.

In conclusion, this case is a reminder of the complication of HLH when encountering patients with disseminated TB accompanied by unexplainable high fever, organomegaly, cytopenia, consumptive coagulopathy, hypertriglyceridemia, and hyperferritinemia. Timely administration of ATT combined with corticosteroids and/or immunoregulation under careful monitoring may achieve a favorable outcome.

Although, it’s difficult for a single case report to thoroughly explore such complicated clinical scenarios which invariably have a fatal outcome, continuous data collection is the need of the hour.

## Data Availability Statement

The raw data supporting the conclusions of this article will be made available by the authors, without undue reservation.

## Ethics Statement

Ethical review and approval was not required for the study on human participants in accordance with the local legislation and institutional requirements. The patients/participants provided their written informed consent to participate in this study.

## Author Contributions

Y-FS, X-HS and YZ analyzed the data, designed the report, and drafted the manuscript. J-XC collected the data, reviewed the literature, and contributed to manuscript drafting. W-XL performed the hematological analyses. J-ML, J-HB, and Y-HW made contribution to clinical management of the patient. J-NC performed the pathologic analyses and guided writing of the manuscript. B-QW was the leader of the team and was responsible for the revision of the manuscript for important content. All authors contributed to the article and approved the submitted version.

## Funding

This work was supported by the Science and Technology Planning Project of Guangdong Province of China, NO.2017A020215177. The funders had no role in study design, collection, analysis, interpretation of data and in writing the manuscript.

## Conflict of Interest

The authors declare that the research was conducted in the absence of any commercial or financial relationships that could be construed as a potential conflict of interest.

## References

[1] Henter JI Horne A Arico M Egeler RM Filipovich AH Imashuku S. Hlh-2004: Diagnostic and Therapeutic Guidelines for Hemophagocytic Lymphohistiocytosis. Pediatr Blood Cancer (2007) 48(2):124–31. 10.1002/pbc.21039 16937360

[2] Al-Samkari H Berliner N. Hemophagocytic Lymphohistiocytosis. Annu Rev Pathol (2018) 13:27–49. 10.1146/annurev-pathol-020117-043625 28934563

[3] Lerolle N Laanani M Riviere S Galicier L Coppo P Meynard JL. Diversity and Combinations of Infectious Agents in 38 Adults With An Infection-Triggered Reactive Haemophagocytic Syndrome: A Multicenter Study. Clin Microbiol Infect (2016) 22(3):268.e1–8. 10.1016/j.cmi.2015.11.018 26686809

[4] Padhi S Ravichandran K Sahoo J Varghese RG Basheer A. Hemophagocytic Lymphohistiocytosis: An Unusual Complication in Disseminated Mycobacterium Tuberculosis. Lung India (2015) 32(6):593–601. 10.4103/0970-2113.168100 26664166 PMC4663863

[5] Dilber E Erduran E Kalyoncu M Aynaci FM Okten A Ahmetoglu A. Hemophagocytic Syndrome as an Initial Presentation of Miliary Tuberculosis Without Pulmonary Findings. Scand J Infect Dis (2002) 34(9):689–92. 10.1080/00365540210147840 12374364

[6] La Rosée P Horne A Hines M von Bahr Greenwood T Machowicz R Berliner N. Recommendations for the Management of Hemophagocytic Lymphohistiocytosis in Adults. Blood (2019) 133(23):2465–77. 10.1182/blood.2018894618 30992265

[7] Lerolle N Laanani M Galicier L Riviere S Meynard JL Azoulay E. Factors Associated With Tuberculosis-Associated Haemophagocytic Syndrome: A Multicentre Case-Control Study. Int J Tuberc Lung Dis (2020) 24(1):124–30. 10.5588/ijtld.19.0856 32005316

[8] Dalugama C Gawarammana IB. Fever With Pancytopenia: Unusual Presentation of Extrapulmonary Tuberculosis: A Case Report. J Med Case Rep (2018) 12(1):58. 10.1186/s13256-018-1596-0 29506574 PMC5838939

[9] Nahid P Dorman SE Alipanah N Barry PM Brozek JL Cattamanchi A. Official American Thoracic Society/Centers for Disease Control and Prevention/Infectious Diseases Society of America Clinical Practice Guidelines: Treatment of Drug-Susceptible Tuberculosis. Clin Infect Dis (2016) 63(7):e147–e95. 10.1093/cid/ciw376 PMC6590850 27516382

[10] Satta G Lipman M Smith GP Arnold C Kon OM McHugh TD. Mycobacterium Tuberculosis and Whole-Genome Sequencing: How Close are We to Unleashing its Full Potential? Clin Microbiol Infect (2018) 24(6):604–9. 10.1016/j.cmi.2017.10.030 29108952

[11] Shi CL Han P Tang PJ Chen MM Ye ZJ Wu MY. Clinical Metagenomic Sequencing for Diagnosis of Pulmonary Tuberculosis. J Infect (2020) 81(4):567–74. 10.1016/j.jinf.2020.08.004 32768450

[12] Miao Q Ma Y Wang Q Pan J Zhang Y Jin W. Microbiological Diagnostic Performance of Metagenomic Next-Generation Sequencing When Applied to Clinical Practice. Clin Infect Dis (2018) 67(suppl_2):S231–S40. 10.1093/cid/ciy693 30423048

[13] Jolobe OMP. Hematological “Red Flags” for Disseminated Tuberculosis: A Diagnostic Opportunity for the Emergency Physician? Am J Emerg Med (2019) 37(6):1195–96. 10.1016/j.ajem.2018.10.027 30340990

[14] Knaak C Nyvlt P Schuster FS Spies C Heeren P Schenk T. Hemophagocytic Lymphohistiocytosis in Critically Ill Patients: Diagnostic Reliability of HLH-2004 Criteria and Hscore. Crit Care (2020) 24(1):244. 10.1186/s13054-020-02941-3 32448380 PMC7245825

[15] Zhang J Wang Y Wu L Wang J Tang R Li S. Application of an Improved Flow Cytometry-Based Nk Cell Activity Assay in Adult Hemophagocytic Lymphohistiocytosis. Int J Hematol (2017) 105(6):828–34. 10.1007/s12185-017-2195-3 28185204

[16] Tucci F Gallo V Barzaghi F Ferrua F Migliavacca M Calbi V. Emapalumab Treatment in an ADA-SCID Patient With Refractory Hemophagocytic Lymphohistiocytosis-Related Graft Failure and Disseminated Bacillus Calmette-Guerin Infection. Haematologica (2021) 106(2):641–6. 10.3324/haematol.2020.255620 PMC7849754 32817285

[17] Park HS Kim DY Lee JH Lee JH Kim SD Park YH. Clinical Features of Adult Patients With Secondary Hemophagocytic Lymphohistiocytosis From Causes Other Than Lymphoma: An Analysis of Treatment Outcome and Prognostic Factors. Ann Hematol (2012) 91(6):897–904. 10.1007/s00277-011-1380-3 22147006

[18] Goto S Aoike I Shibasaki Y Morita T Miyazaki S Shimizu T. A Successfully Treated Case of Disseminated Tuberculosis-Associated Hemophagocytic Syndrome and Multiple Organ Dysfunction Syndrome. Am J Kidney Dis (2001) 38(4):E19. 10.1053/ajkd.2001.27727 11576906

[19] Seminari E Contardi G Rubert L Fronti E Comoli P Minoli L. Tuberculosis-Induced Haemophagocytic Syndrome in a Patient on Haemodialysis Treated With Anti-Thymocyte Globulin. Int J Tuberc Lung Dis (2014) 18(2):248–9. 10.5588/ijtld.13.0533 24429321

[20] Sarathy J Blanc L Alvarez-Cabrera N O’Brien P Dias-Freedman I Mina M. Fluoroquinolone Efficacy Against Tuberculosis is Driven by Penetration Into Lesions and Activity Against Resident Bacterial Populations. Antimicrob Agents Chemother (2019) 63(5):e02516–8. 10.1128/AAC.02516-18 PMC6496041 30803965

[21] Critchley JA Young F Orton L Garner P. Corticosteroids for Prevention of Mortality in People With Tuberculosis: A Systematic Review and Meta-Analysis. Lancet Infect Dis (2013) 13(3):223–37. 10.1016/S1473-3099(12)70321-3 23369413

[22] Brastianos PK Swanson JW Torbenson M Sperati J Karakousis PC. Tuberculosis-Associated Haemophagocytic Syndrome. Lancet Infect Dis (2006) 6(7):447–54. 10.1016/S1473-3099(06)70524-2 16790385

[23] Singha A Mukherjee A Dasgupta R Das T. A Case of Hemophagocytic Syndrome Due to Tuberculosis: Uncommon Manifestation of a Common Disease. Case Rep Med (2014) 2014:613845. 10.1155/2014/613845 25404945 PMC4227369

[24] Rathnayake PV Kularathne WK De Silva GC Athauda BM Nanayakkara SN Siribaddana A. Disseminated Tuberculosis Presenting as Hemophagocytic Lymphohistiocytosis in an Immunocompetent Adult Patient: A Case Report. J Med Case Rep (2015) 9:294. 10.1186/s13256-015-0772-8 26714642 PMC4696226

[25] Ranjan A Pal RS Kumar A Chandra Ojha U. Haemophagocytic Lymphohistiocytosis (Hlh) Secondary to Miliary Tuberculosis. Indian J Tuberc (2020) 67(3):366–70. 10.1016/j.ijtb.2019.08.006 32825870

[26] Trovik LH Sandnes M Blomberg B Holmaas G Ahmed AB Tvedt THA. Hemophagocytic Lymphohistiocytosis and Miliary Tuberculosis in a Previously Healthy Individual: A Case Report. J Med Case Rep (2020) 14(1):217. 10.1186/s13256-020-02555-x 33172493 PMC7655140

[27] Shi W Jiao Y. Nontuberculous Mycobacterium Infection Complicated With Haemophagocytic Syndrome: A Case Report and Literature Review. BMC Infect Dis (2019) 19(1):399. 10.1186/s12879-019-4061-9 31072325 PMC6507031

[28] Naha K Dasari S Vivek G Prabhu M. Disseminated Tuberculosis Presenting With Secondary Haemophagocytic Lymphohistiocytosis and Poncet’s Disease in an Immunocompetent Individual. BMJ Case Rep (2013) 2013:bcr2012008265. 10.1136/bcr-2012-008265 PMC3644889 23563676

[29] Kessler M Reinig E. Hlh Associated With Disseminated Tuberculosis. N Engl J Med (2020) 382(18):1749. 10.1056/NEJMicm1910558 32348646

[30] Parsi M Dargan K. Hemophagocytic Lymphohistiocytosis Induced Cytokine Storm Secondary to Human Immunodeficiency Virus Associated Miliary Tuberculosis. Cureus (2020) 12(1):e6589. 10.7759/cureus.6589 32051801 PMC7001132

[31] Jaime-Perez JC Aguilar-Calderon PE Salazar-Cavazos L Gomez-Almaguer D. Evans Syndrome: Clinical Perspectives, Biological Insights and Treatment Modalities. J Blood Med (2018) 9:171–84. 10.2147/JBM.S176144 PMC6190623 30349415

[32] Audia S Grienay N Mounier M Michel M Bonnotte B. Evans’ Syndrome: From Diagnosis to Treatment. J Clin Med (2020) 9(12):3851. 10.3390/jcm9123851 PMC7759819 33260979

[33] Asaji M Tobino K Murakami K Goto Y Sueyasu T Nishizawa S. Miliary Tuberculosis in a Young Woman With Hemophagocytic Syndrome: A Case Report and Literature Review. Intern Med (2017) 56(12):1591–6. 10.2169/internalmedicine.56.8025 PMC5505920 28626190

