# Supplementary material for: Disseminated Tuberculosis Associated Hemophagocytic Lymphohistiocytosis in a Pregnant Woman With Evans syndrome: A Case Report and Literature Review
Source: Front Immunol. 2021 Jun 10;12:676132. doi: 10.3389/fimmu.2021.676132 (PMC8222916; doi:10.3389/fimmu.2021.676132)
Supplement: Supplementary file 1 [file DataSheet_1.pdf]

Supplementary file 1

Immunological profile

Items of autoimmune indicator	Result
Anti-nuclear antibody	Negative
Anti double-strain DNA antibody	Negative
Anti histone antibody	Negative
Anti c1q antibody	Negative
Anti smith antibody	Negative
Anti SSA antibody	Negative
Anti SSB antibody	Negative
Anti u1rnp antibody	Negative
Anti Jo-1 antibody	Negative
Anti Smith antibody	Negative
Anti SCL-70 antibody	Negative
Anti r-RNP antibody	Negative
Anti Ku antibody	Negative
Anti PM-1 antibody	Negative
Anti-neutrophilic cytoplasmic antibody (P-ANCA, C-ANCA, PMO-ANCA, PR3-ANCA)	Negative
Anti cardiolipin antibody	Negative
Coomb’s test	Negative
