# Supplementary material for: Disseminated Tuberculosis Associated Hemophagocytic Lymphohistiocytosis in a Pregnant Woman With Evans syndrome: A Case Report and Literature Review
Source: Front Immunol. 2021 Jun 10;12:676132. doi: 10.3389/fimmu.2021.676132 (PMC8222916; doi:10.3389/fimmu.2021.676132)
Supplement: Supplementary file 2 [file DataSheet_2.pdf]

Supplementary file 2

Procedure, quality control and results of mNGS

1. Procedure of mNGS

Bronchoalveolar lavage fluid (BALF) and blood were collected independently. The samples were stored in a sterile container, then were preserved and were transported in dry ice. The whole process of sequencing and pathogen detection pipeline were carried out in the laboratory of Guangzhou Kingmed Medical Test Center Co., Ltd.

The nucleic acid of samples was extracted by QIAsymphony Circulating NA Kit (Cus.48), together with a negative control and a positive control. Next, the DNA and cDNA libraries were prepared using TruePrepTM DNA Library Prep Kit V2 for Illumina® (Vazyme Biotech Co., Ltd，TD503). The DNA libraries concentration was measured by Qubit. Then the sequencing was carried out by Illumina nextseq 500 system with 75 cycles Reagent Kit. High-quality sequencing data were obtained by filtering out reads of low-quality. Then, reads of human were removed by mapping reads to human reference genome. The remaining data were aligned to the microbial genome database.

The procuture of filtering, mapping and alignment are carriyed out by the widely used software Sequence-Based Ultra-Rapid Pathogen Identification (SURPI), which is generally considered to be highly accurate. The microbial genome database is MetagenomicX for clinical application. The whole database contains 22934 microorganisms, which covers most of the microbial genomes which have been sequenced. 8704 of the total microorganisms in the first-grade database have integral sequence of whole genome and detailed clinical analysis, which covers most of the known pathogenic bacteria, viruses, fungi and parasites. This first-grade database with high-quality genomes is used as first choice.

2. Quality control of procedure

During the process of sampling, storage, transportation and sequencing, aseptic procedures are strictly followed to ensure that qualified BALF and blood specimens are collected. In the lab, the specimens were kept in the -80 degree refrigerator to prevent nucleic acid degradation. All detection procedures are finished within 48 hours after receiving specimen ensuring timely guidance for clinical treatment.

Both Nucleic acid extraction and library preparation were conducted in parallel with quality control samples. And we compared the results of this whole process with the results analysed

by software of Burrows Wheeler Alignment (BWA). The results of the two pipelines are highly consistent. To eliminate background interference, a minimum threshold of 10 RPM-r (RPM defined as Reads per million, RPM-r defined as RPMsample/RPMno-template-control) is designated for reporting the detection of a microorganism as “detected”.

3. Results and quality control of mNGS in this case

3.1 mNGS of the BALF identified 926 DNA sequence reads and 195 RNA sequence reads corresponding to M. tuberculosis complex (MTBC), respectively. MTBC contains 5 species: *Mycobacterium tuberculosis, Mycobacterium canettii, Mycobacterium decipiens, Mycobacterium mungi and Mycobacterium orygis.* For these two DNA and RNA mNGS of BALF, 96.71% and 95.71% reads were filtered for human genome respectively. And 225958 reads and 491668 reads were mapped to the microbial genomes database respectively. The quality scores across all bases in the mNGS of BALF were showed as the figures below.

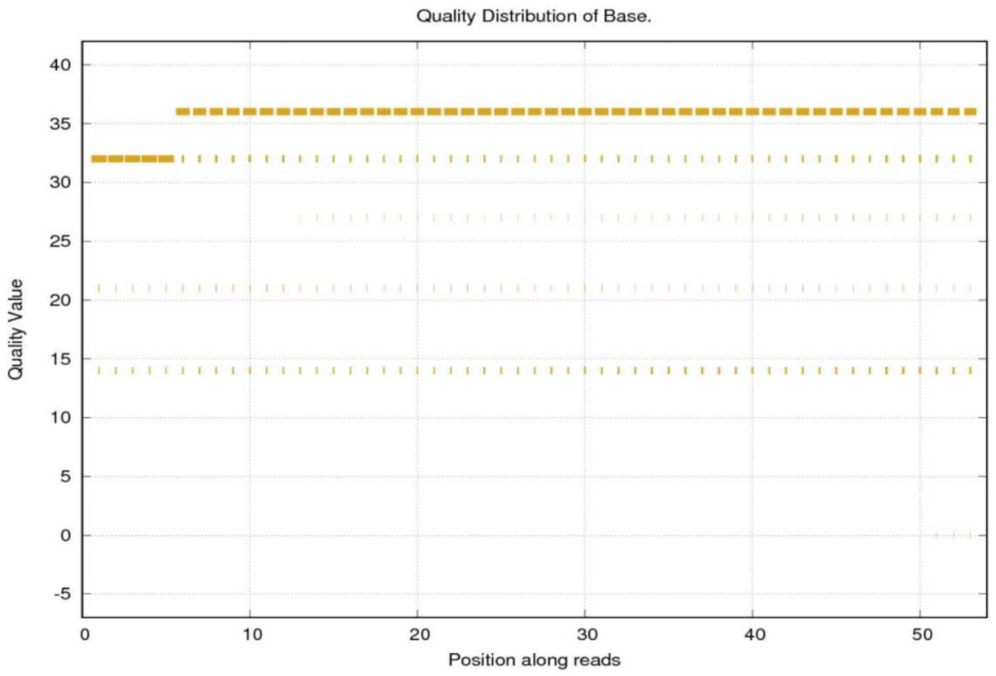

Fig.1 The quality scores across all bases in the DNA mNGS of BALF

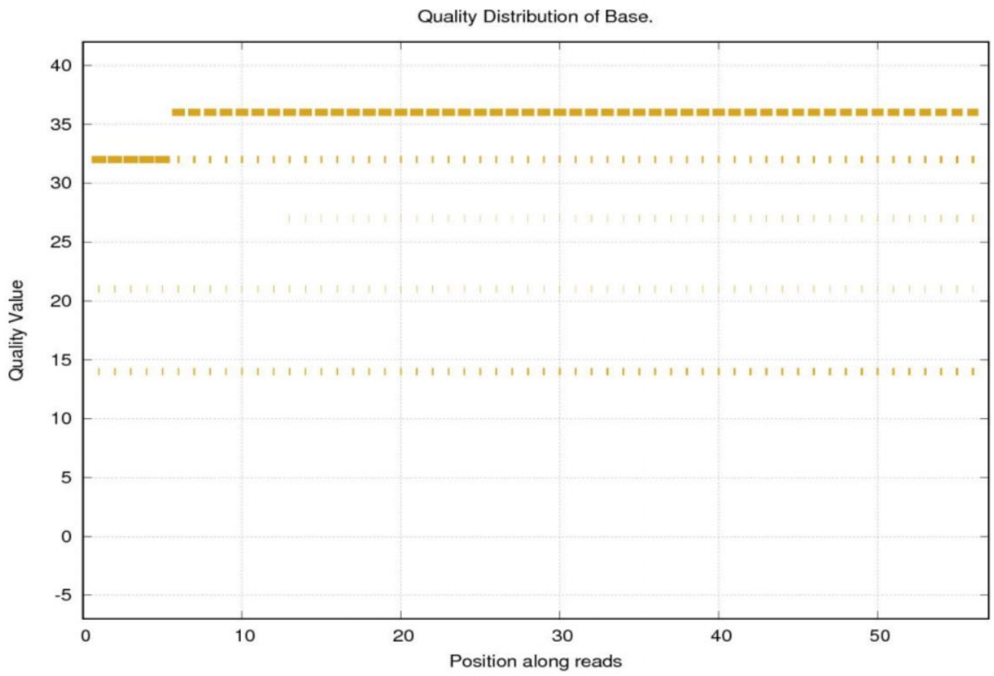

Fig. 2 The quality scores across all bases in the RNA mNGS of BALF.

3.2 mNGS of the blood identified 48 DNA sequence reads of M. tuberculosis. 99.49% reads were filtered for human genome respectively. And 125859 reads were mapped to the microbial genomes database. The quality scores across all bases in the mNGS and sequencing coverage were showed as the figures below.

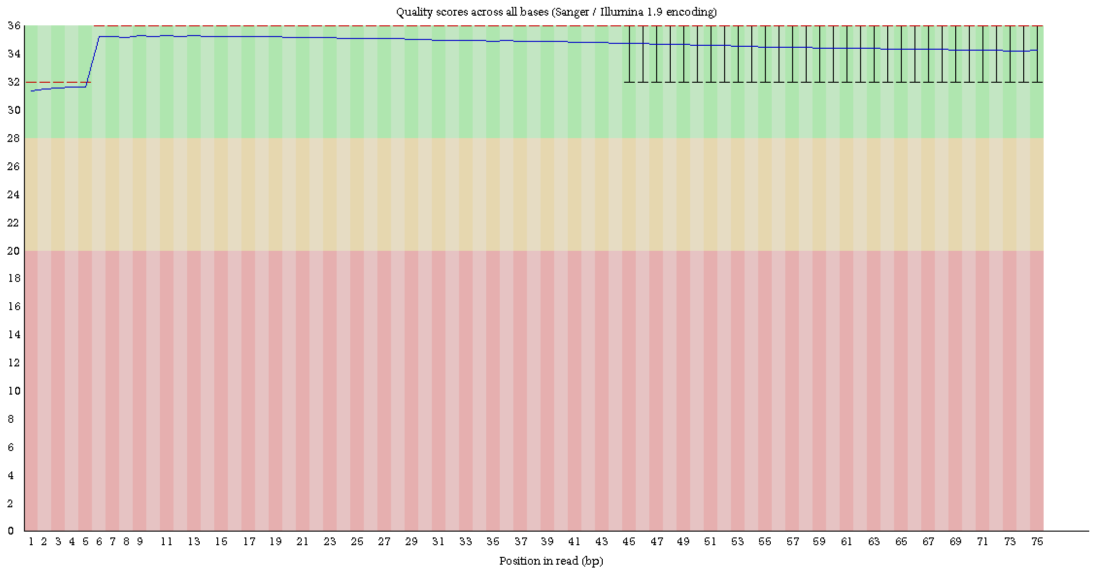

Fig.3 The quality scores across all bases in the DNA mNGS of Blood.

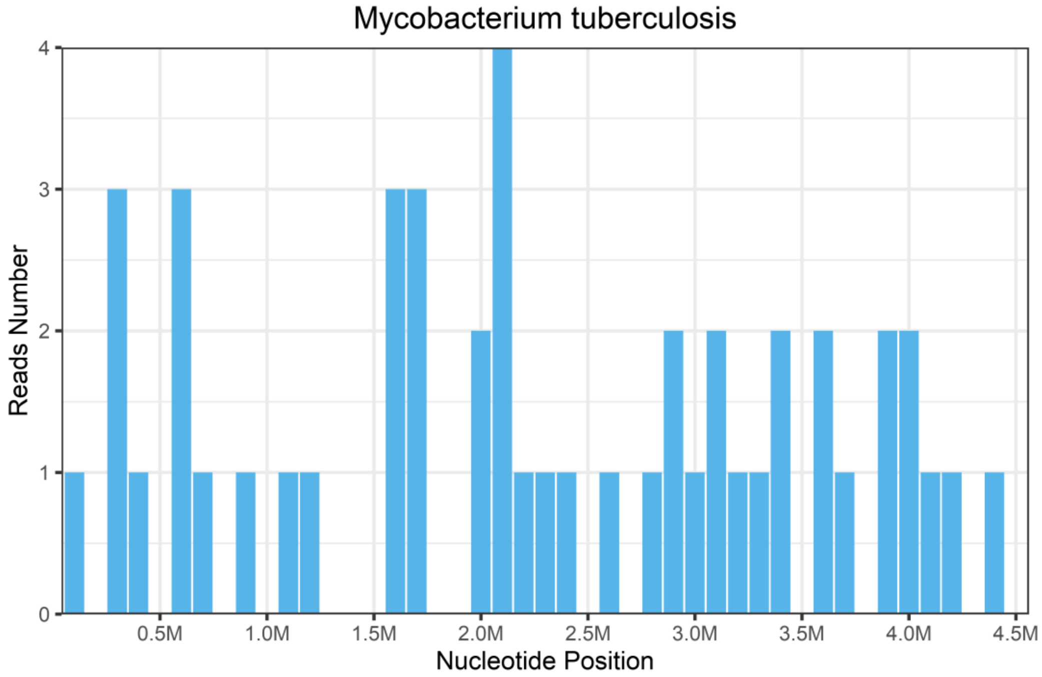

Fig.4 The Sequencing coverage in the DNA mNGS of Blood.
