# Supplementary material for: Disseminated Tuberculosis Associated Hemophagocytic Lymphohistiocytosis in a Pregnant Woman With Evans syndrome: A Case Report and Literature Review
Source: Front Immunol. 2021 Jun 10;12:676132. doi: 10.3389/fimmu.2021.676132 (PMC8222916; doi:10.3389/fimmu.2021.676132)
Supplement: Supplementary file 3 [file DataSheet_3.pdf]

Genetic testing for primary HLH

This genetic testing is based on a mNGS panel which covers 62 genes (shown in the table below) related to primary HLH or previously reported in HLH cases. Single nucleotide variants, copy number variants and Indel in each exon of these genes were routinely detected.

Abbreviation of gene	Full Name
ADA	ADENOSINE DEAMINASE
AK2	ADENYLATE KINASE 2
AP3B1	ADAPTOR-RELATED PROTEIN COMPLEX 3, BETA-1 SUBUNIT
ATM	ATM SERINE/THREONINE KINASE
BLM	BLOOM SYNDROME
BLOC1S6	BIOGENESIS OF LYSOSOME-RELATED ORGANELLES COMPLEX 1, SUBUNIT 6
CARD11	CASPASE RECRUITMENT DOMAIN-CONTAINING PROTEIN 11
CASP10	CASPASE 10, APOPTOSIS-RELATED CYSTEINE PROTEASE
CASP8	CASPASE 8, APOPTOSIS-RELATED CYSTEINE PROTEASE
CD27	CD27 ANTIGEN
CD40LG	CD40 LIGAND
COG1	COMPONENT OF OLIGOMERIC GOLGI COMPLEX 1
COG6	COMPONENT OF OLIGOMERIC GOLGI COMPLEX 6
CORO1A	CORONIN 1A
DCLRE1C	DNA CROSS-LINK REPAIR PROTEIN 1C
DKC1	DYSKERIN
DNMT3B	DNA METHYLTRANSFERASE 3B
DOCK8	DEDICATOR OF CYTOKINESIS 8
FADD	FAS-ASSOCIATED VIA DEATH DOMAIN
FAS	FAS CELL SURFACE DEATH RECEPTOR
FASLG	FAS LIGAND
FCGR3A	Fc FRAGMENT OF IgG RECEPTOR IIIa
GATA2	GATA-BINDING PROTEIN 2
IL21R	INTERLEUKIN 21 RECEPTOR
IRF8	INTERFERON REGULATORY FACTOR 8
ITK	IL2-INDUCIBLE T-CELL KINASE
JAK3	JANUS KINASE 3
LRBA	LIPOPOLYSACCHARIDE-RESPONSIVE, BEIGE-LIKE ANCHOR PROTEIN
LYST	LYSOSOMAL TRAFFICKING REGULATOR
MAGT1	MAGNESIUM TRANSPORTER 1
MCM4	MINICHROMOSOME MAINTENANCE COMPLEX COMPONENT 4
MVK	MEVALONATE KINASE
MYO5A	MYOSIN VA
NCF1	NEUTROPHIL CYTOSOLIC FACTOR 1
NCF2	NEUTROPHIL CYTOSOLIC FACTOR 2
NCF4	NEUTROPHIL CYTOSOLIC FACTOR 4
NLRC4	NLR FAMILY, CASPASE RECRUITMENT DOMAIN-CONTAINING 4

NLRP12	NLR FAMILY, PYRIN DOMAIN-CONTAINING 12
OSTM1	OSTEOPETROSIS-ASSOCIATED TRANSMEMBRANE PROTEIN 1
PIK3CD	PHOSPHATIDYLINOSITOL 3-KINASE, CATALYTIC, DELTA
PIK3R1	PHOSPHATIDYLINOSITOL 3-KINASE, REGULATORY SUBUNIT 1
PLCG2	PHOSPHOLIPASE C, GAMMA-2
PRF1	PERFORIN 1
PRKCD	PROTEIN KINASE C, DELTA
RAB27A	RAS-ASSOCIATED PROTEIN RAB27A
RAG1	RECOMBINATION-ACTIVATING GENE 1
RAG2	RECOMBINATION-ACTIVATING GENE 2
RECQL4	RECQ PROTEIN-LIKE 4
SH2D1A	SH2 DOMAIN PROTEIN 1A
SH3BP2	SH3 DOMAIN-BINDING PROTEIN 2
SLC29A3	SOLUTE CARRIER FAMILY 29 (NUCLEOSIDE TRANSPORTER), MEMBER 3
SLC7A7	SOLUTE CARRIER FAMILY 7 (CATIONIC AMINO ACID TRANSPORTER, y+ SYSTEM), MEMBER 7
STX11	SYNTAXIN 11
STXBP2	SYNTAXIN-BINDING PROTEIN 2
TCIRG1	T CELL IMMUNE REGULATOR 1
TNFRSF11A	TUMOR NECROSIS FACTOR RECEPTOR SUPERFAMILY, MEMBER 11A
TNFRSF13B	TUMOR NECROSIS FACTOR RECEPTOR SUPERFAMILY, MEMBER 13B
UNC13D	UNC13 HOMOLOG D
UNG	URACIL-DNA GLYCOSYLASE
XIAP	INHIBITOR OF APOPTOSIS, X-LINKED
ZAP70	ZETA-CHAIN-ASSOCIATED PROTEIN KINASE

The abbreviation and full name of the genes were confirmed on https://omim.org.
